# Genome Sequences of 19 *Rhodococcus erythropolis* Cluster CA Phages

**DOI:** 10.1128/genomeA.01201-17

**Published:** 2017-12-07

**Authors:** J. Alfred Bonilla, Sharon Isern, Ann M. Findley, Karen K. Klyczek, Scott F. Michael, Margaret S. Saha, William J. Buchser, Mark H. Forsyth, Sudip Paudel, Christopher R. Gissendanner, Allison M. D. Wiedemeier, Fernanda L. Alonzo, University of Wisconsin–River Falls SEA-PHAGES, Florida Gulf Coast University SEA-PHAGES, University of Louisiana–Monroe SEA-PHAGES, College of William & Mary SEA-PHAGES, Rebecca A. Garlena, Daniel A. Russell, Welkin H. Pope, Steven G. Cresawn, Deborah Jacobs-Sera, Graham F. Hatfull

**Affiliations:** a Department of Biology, University of Wisconsin–River Falls, River Falls, Wisconsin, USA; b Department of Biological Sciences, Florida Gulf Coast University, Fort Myers, Florida, USA; c Biology Program, School of Sciences, University of Louisiana–Monroe, Monroe, Louisiana, USA; d Department of Biology, College of William & Mary, Williamsburg, Virginia, USA; e Department of Biological Sciences, University of Pittsburgh, Pittsburgh, Pennsylvania, USA; f Department of Biology, James Madison University, Harrisonburg, Virginia, USA

## Abstract

We report the complete genome sequences of 19 cluster CA bacteriophages isolated from environmental samples using *Rhodococcus erythropolis* as a host. All of the phages are *Siphoviridae*, have similar genome lengths (46,314 to 46,985 bp) and G+C contents (58.5 to 58.8%), and share nucleotide sequence similarity.

## GENOME ANNOUNCEMENT

A large collection of sequenced mycobacteriophages reveals substantial genetic diversity and mosaic genomic architectures (1). Smaller collections of phages have been isolated on other hosts within the phylum *Actinobacteria*, including *Rhodococcus* spp. (2). Previously described *Rhodococcus* phages include DocB7, Pepy6, Pine5, Poco6, REQ1, REQ2, REQ3, and E3 isolated on *R. equi*, and RER2, RGL3, and RRH1 isolated on *R. erythropolis*, *R. globerulus*, and *R. rhodochrous*, respectively (3–7), representing nine different genome types (2).

Students in the Howard Hughes Medical Institute (HHMI) Science Education Alliance–Phage Hunters Advancing Genomics and Evolutionary Science (SEA-PHAGES) program (8) isolated phages infecting *R. erythropolis* RIA 643 using environmental samples from diverse geographical locations (Table 1). Following purification and amplification, genomic DNA was isolated and sequenced using Illumina MiSeq 150-base reads and assembled with Newbler and Consed to major contigs with a 150-fold minimum coverage. Genomes were annotated using DNA Master (http://cobamide2.bio.pitt.edu/), Glimmer (9), GeneMark (10), ARAGORN (11), and tRNAscan-SE (12). Functions were assigned using BLASTp (13), HHpred (14), and Phamerator (15). The 19 genomes have similar lengths (46,314 to 46,985 bp) and G+C contents (58.5 to 58.8%), and have defined genome ends with 10-base 3′ single-stranded extensions (5′-CGGCCGTGAT). All share nucleotide sequence similarity (>93% pairwise DNA identity) with each other and with phages RER2 and RGL3 (7), and are grouped in cluster CA. CosmicSans, Lillie, Rhodalysa, and TWAMP were isolated from distinct but geographically similar locations and have similar genomes, differing by 2 to 6 nucleotides.

**TABLE 1:** Cluster CA phages of *Rhodococcus erythropolis*

Phage	GenBank accession no.	Genome size (bp)	G+G content (%)	No. of PCGs^a^	Location of isolation
Alatin	MF324905	46,673	58.7	65	Hudson, WI
Angryorchard	KY549153	46,597	58.8	64	Somerville, TN
AppleCloud	MF324903	46,389	58.7	65	Hudson, WI
BobbyDazzler	KY549154	46,641	58.8	65	Monroe, LA
Bonanza	MF537628	46,932	58.8	66	Keithville, LA
CosmicSans	KT372002	46,596	58.5	66	Williamsburg, VA
Harlequin	KX611788	46,383	58.8	66	Monroe, LA
Hiro	MF324898	46,854	58.7	65	Boston, MA
Jester	MF373842	46,314	58.7	66	Ouachita Parish, LA
Krishelle	MF324902	46,985	58.5	67	Hudson, WI
Lillie	KT990218	46,596	58.6	66	Williamsburg, WI
Naiad	MF324901	46,619	58.6	65	Hudson, WI
Natosaleda	KX550082	46,527	58.6	65	Boston, MA
Partridge	KX712237	46,962	58.8	66	Monroe, LA
RexFury	MF324904	46,627	58.6	65	River Falls, WI
Rhodalysa	KT375356	46,596	58.5	66	Williamsburg, VA
StCroix	MF324900	46,619	58.6	65	Hudson, WI
TWAMP	KT959213	46,596	58.5	66	Williamsburg, VA
Yogi	KX712236	46,930	58.8	66	Richland Parish, LA

a PCGs = protein-coding genes.

We identified 64 to 67 predicted protein-coding genes in each genome, of which ~50% were ascribed putative functions, as well as 2 to 3 tRNA genes located approximately 1.5 kbp from the left genome end. The genome architectures are reminiscent of the cluster A mycobacteriophages such as L5 (16), with the virion structure and assembly genes transcribed rightward in the left arms and regulatory and replication genes transcribed leftward in the right arms. All of the cluster CA *Rhodococcus* phages are predicted to be temperate and encode putative immunity repressors (e.g., Alatin gp57) and serine integrases (e.g., Alatin gp29) close to the genome center; the chromosomal integration site is not known. Stable lysogens have been isolated for several of the phages, and all of those tested are homoimmune.

Although these *Rhodococcus* phages do not share extensive nucleotide sequence similarity to the cluster A mycobacteriophages, over 40% of the predicted gene products have amino acid sequence similarity to cluster A gene products. They likely also share a regulatory system with cluster A phages in which the immunity repressor binds to multiple short (13-bp) asymmetric repeats (operators and stoperators) located in small intergenic gaps (17, 18) preventing lytic gene transcription in the prophage. The cluster CA *Rhodococcus* phages each have up to 20 copies of repeated sequences related to the 13-bp consensus 5′-TGTCTATTGTCAA, positioned in intergenic spaces and primarily in one orientation with respect to the direction of transcription. This array of features shared between cluster A mycobacteriophages and cluster CA *Rhodococcus* phages suggests they may warrant inclusion in a higher taxonomic level, a “supercluster” (19).

### Accession number(s).

The 19 *Rhodococcus erythropolis* phage genome sequences are available in GenBank with accession numbers as shown in Table 1.
